# Oxidative Stress in Schizophrenia

**DOI:** 10.2174/157015911795596595

**Published:** 2011-06

**Authors:** Marija Bošković, Tomaž Vovk, Blanka Kores Plesničar, Iztok Grabnar

**Affiliations:** 1 Faculty of Pharmacy, University of Ljubljana, Aškerčeva 7, 1000 Ljubljana, Slovenia; 2 University Psychiatric Clinic Ljubljana, Studenec 48, 1260 Ljubljana, Slovenia

**Keywords:** Schizophrenia, oxidative stress, antioxidants, fatty acids, biochemical markers, tardive dyskinesia, antipsychotics.

## Abstract

Increasing evidence indicates that oxidative damage exists in schizophrenia. Available literature about possible mechanisms of oxidative stress induction was reviewed. Furthermore, possibilities of measuring biomarkers of schizophrenia outside the central nervous system compartment, their specificity for different types of schizophrenia and potential therapeutic strategies to prevent oxidative injuries in schizophrenia were discussed. Data were extracted from published literature found in Medline, Embase, Biosis, Cochrane and Web of Science, together with hand search of references. Search terms were: schizophrenia, oxidative stress, antipsychotics, antioxidants and fatty acids. Finding a sensitive, specific and non invasive biomarker of schizophrenia, which could be measured in peripheral tissue, still stays an important task. Antioxidant enzymes, markers of lipid peroxidation, oxidatively modified proteins and DNA are most commonly used. As it considers the supplemental therapy, according to our meta-analysis vitamin E could potentially improve tardive dyskinesia, while for the effect of therapy with polyunsaturated fatty acids there is no clear evidence. Oxidative stress is a part of the pathology in schizophrenia and appears as a promising field to develop new therapeutic strategies. There is a need for well designed, placebo controlled trials with supplementation therapy in schizophrenia.

## INTRODUCTION

Schizophrenia is a devastating mental disorder, expressed in the form of abnormal mental functions and disturbed behavior. It has a life-time prevalence of approximately 1% of the world’s population [1]. The disorder has a bad outcome, regardless of different treatments (pharmacological, social, cognitive etc.). Cardinal symptoms of schizophrenia include positive and negative symptoms, and cognitive dysfunction and deterioration in social and occupational functioning [2]. Schizophrenia is related to different neurodevelopmental, structural and behavioral abnormalities. It has been proposed that such abnormalities could originate from malfunctioning genes and/or non-genetic factors such as ethnicity, drug and alcohol abuse, life style, medications, pre-natal and neonatal infections, maternal malnutrition, complications during birth and many other factors. By inducing cellular metabolic stress these factors appear to increase the possibility of oxidative stress and damage [3, 4]. Oxidative stress is common to several psychiatric disorders [5]. The mechanisms have been most widely studied in schizophrenia, employing various areas of oxidative research, including oxidative biomarkers assays, psychopharmacology, and clinical studies with antioxidants.

The scope of this article is to review the available literature on oxidative status in patients with schizophrenia, including reactive oxygen species and reactive nitrogen species production, antioxidant defense and influence of antipsychotic treatment. Furthermore, the possibility of measuring biomarkers in different tissues, their specificity for different types of schizophrenia, and potential therapeutic strategies to prevent reactive species (RS) mediated injuries in patients with schizophrenia will be examined. Data was obtained by searching the published literature: Medline, Embase, Biosis, Cochrane and Web of Science. Search terms were: schizophrenia, oxidative stress, antipsychotics, antioxidants, and fatty acids.

## METHODOLOGICAL CONSIDERATIONS

### Oxidative Stress

Oxidative stress is defined as a disturbance in the prooxidant/antioxidant balance in favor of the former, leading to potential damage. Thus, diminished antioxidants and/or increased production of RS will result in oxidative damage of cell lipids, proteins, enzymes, carbohydrates and DNA [6]. A growing body of evidence indicates that oxidative damage exists in schizophrenia [7-9]. Although this may not be the main cause, oxidative damage has been suggested to be a common pathogenic process that contributes to declining course and poor outcome in schizophrenia [7, 10-13].

Cells in the central nervous system (CNS) are more vulnerable to the toxic effects of RS than those in other organs of the body. Brain has a high rate of oxidative metabolic activity, high oxygen consumption, low levels of protective antioxidant enzymes, a high ratio of membrane surface area to cytoplasmic volume, and a neuronal anatomical network vulnerable to disruption. The high proportion of readily oxidizable membrane polyunsaturated fatty acids (PUFAs) make it more exposed to oxidative stress [7]. Additionally, auto-oxidizable neurotransmitters, like dopamine (DA), epinephrine and norepinephrine, are present in excess in the brain. Metabolism of neurotransmitters generates large amounts of hydrogen peroxide (H_2_O_2_) and neuronal mitochondria can generate superoxide radical (O_2_^•-^) [14]. Furthermore, the presence of potentially toxic amino acids can trigger cell proteolysis. Of all the brain regions, the basal ganglia may be at particular risk for RS induced damage due to their large content of iron [7, 14-16].

### Antioxidative Defense

Human body has a complex defense system of antioxidant enzymes, including superoxide dismutase (SOD), glutathione peroxidase (GpX), and catalase (CAT). These enzymes block the initiation of RS chain reactions [17]. The non-enzymatic antioxidant components are compounds such as glutathione (GSH), vitamin E, vitamin C and β-carotene, which react with RS and thereby prevent the propagation of chain reactions [14, 18, 19]. Oxidative damage of the brain is suggested by increased lipid peroxidation products in the cerebrospinal fluid and plasma, and reduced membrane PUFAs in the brain and red blood cell (RBC) membranes [11]. In response to increased RS formation and related membrane damage due to lipid peroxidation, the concentration of antioxidant enzymes can rise as a compensatory mechanism. Due to their specificity and affinity, in addition to their power of self-protection with redox elements in their active site and the possibility of amplification, antioxidant enzymes are the most important defence against RS [20].

### Can Peripheral Biomarkers Reflect Status in the CNS?

Most measurements of oxidative stress in patients with schizophrenia have been made on peripheral tissues. There is a lack of information on oxidative processes in cerebrospinal fluid and brain. It must be stressed that traces of oxidative damage may originate from various sources in the body and consequently, such a peripheral indicator may not necessarily reflect the conditions of the oxidative stress parameters in the brain [12]. Considering the size of the CNS in comparison to the other compartments of the human body, it seems reasonable to assume that changes in the levels of enzymes inside the brain can influence the enzyme plasma levels [21].

RBCs have often been used for evaluation of oxidative stress in patients with schizophrenia. Some illness-related abnormalities in RBCs may reflect equivalent abnormalities in the neurons, which are hard to examine *in vivo* [12, 22-29]. Because of its accessibility, the RBC membrane is commonly used as a “window” into the CNS. There are findings that a deficit identified in the RBC membrane is also present in the brain of patients with schizophrenia [27-29]. Furthermore, alterations in phospholipids metabolism have been shown in post-mortem brain of schizophrenia patients. 31P magnetic resonance spectroscopy allows *in vivo* evaluation of phospholipids metabolism in the brain. Using this noninvasive technique Williamson *et al*. have demonstrated presence of membrane phospholipid abnormalities in schizophrenia, but they could not prove with certainty that these abnormalities are specific to schizophrenia [30]. However, in a later 31P magnetic resonance study conducted on postmortem brain of patients with schizophrenia, compared to control there was no significant difference for any subclass of phospholipids [31].

Significantly lower levels of GSH, GpX and glutathione reductase (GR) were found in the brain of schizophrenia patients than in controls, which correlates with the peripheral measurements [23-26]. On the other hand, there are studies with proton magnetic resonance reporting non significant reduction, or even an increase of GSH concentration in the brain [32-34].

There are also some suggestions that peripheral nitric oxide (NO) metabolites can be used as markers of CNS-dependent NO changes. Serum total nitrite was increased in a group of demyelinating diseases including multiple sclerosis, inflammatory neurological diseases and in AIDS patients [22].

Besides the theory that oxidative status in CNS influences the concentration of peripheral biomarkers, it should be stressed that also the reverse hypothesis is possible. In a study by Aytan *et al*., it has been demonstrated that peripheral oxidative stress can change oxidative status of CNS. In a rabbit model high cholesterol feeding caused an increase in serum malondialdehyde (MDA), which clearly correlated with an increase in protein oxidation parameters in the brain [35].

Due to heterogeneity of empirical evidence, currently there is no clear support to the value of peripheral biomarkers as markers of central oxidative status. It can be concluded that the results of studies on CNS (cerebrospinal fluid, postmortem or animals) [24, 28, 36-38] show at least correlative tendencies with studies assaying biomarkers in erythrocytes, plasma or polymorphonuclears. The use of blood components in examining RS mediated pathology in schizophrenia appears reasonable [39]. Peripheral biomarkers of RS mediated damage have also been suggested to be useful clinically in identifying patients with a high risk of developing different symptoms of schizophrenia or adverse events of antipsychotic treatment such as extrapyramidal syndrome [40].

## ABNORMALITIES OF OXIDATIVE STATUS

### Evaluation of Oxidative Status in Schizophrenia

Antioxidant enzymes such as SOD, GpX and CAT are most commonly measured for quantifying the antioxidative defense in schizophrenia, along with vitamin E and C levels [41, 42]. While the majority of studies have reported decreased antioxidant defense in patients with schizophrenia [4, 10, 13, 21, 26, 42-54] there are also some studies where the opposite has been reported [12, 55-59]. Several factors, such as differences in measuring techniques, differences in material tested, exposure to antipsychotic treatment, sampling of patients at different stages of the disease, differences in disease etiology and ethnic origin, lifestyle and dietary patterns, may be responsible for this discrepancy. Antioxidant enzymes are usually measured in RBCs. According to Halliwell *et al*., antioxidant enzyme levels can drop as a result of RBC ageing processes. Consequently these measurements reflects only RBC turnover in circulation [60]. Additionally, MDA tends to have higher levels in summer compared to winter [61].

Antioxidant enzymes levels may be lower at the very early stages of psychotic disorder, and may further depend on the type of medication, the severity of psychopathology or environmental factors [4]. On the other hand, it was suggested that decreased antioxidant defense probably exists later in patients on chronic treatment with antipsychotics [59]. It was also indicated that haloperidol or other antipsychotics may not have a direct effect on antioxidant enzymes and that severity of symptomatology, may influence their activities, particularly of SOD and GpX [4, 62].

Taken all together, the majority of studies confirm that oxidative stress and oxidative damage is present in schizophrenia, in never-medicated and early stage of disease and in treated and chronic stage as well [4, 10, 13, 21, 26, 42-54, 63].

Considerable attention has been focused on the determination of biomarkers of lipid peroxidation in schizophrenia. PUFAs peroxidation is a chain reaction with a large number of intermediates and end point molecules. It can be assayed in many ways, such as determination of lipid peroxides, isoprostanes, aldehydic end products like MDA and 4-hydroxynonenal (HNE), and by the determination of volatile hydrocarbons. The most frequently assayed marker of lipid peroxidation in schizophrenia are thiobarbituric acid related substances (TBARS). TBARS have been widely criticized for their poor specificity, but they are still acceptable and widely used in researches of lipid peroxidation [64]. Results of the recent meta-analysis [65] do not lessen belief in this parameter, at least within the context of schizophrenia research. Measurements of unsaturated aldehydes, such as HNE and acrolein, together with isoprostanes, exhaled hydrocarbons, and products of enzymatic peroxidation of arachidonic acid, such as tromboxane B2 and its metabolite 11-dehydrotromboxane B2 have been recommended [60, 66, 67].

Determination of oxidatively modified proteins seems to be relevant, considering their importance in cellular function, their critical roles in enzyme catalysis, ligand binding and signal transduction, and since damaged proteins can contribute to secondary damage of other molecules. Oxidative modification of proteins results in new functional groups and can seriously compromise cellular integrity. Protein carbonyls are an accepted index of protein oxidation and levels may be readily quantified in a variety of ways, including an enzyme-linked immunosorbent assay method. They are not specific markers of oxidative damage since bound aldehydes and glycated proteins are also measured [60, 68]. Furthermore, proteins can be damaged by compounds other than oxygen species and give products such as parahydroxyphenylacetaldehyde, 3-chlorotyrosine, 3,5-dichlorotyrosine, 3-bromotyrosine and 3-nitrotyrosine. 3-nitrotyrosine in human plasma proteins has been most commonly assayed [60]. Oxidative modifications of proteins, measured by 3-nitrotyrosine and carbonyl groups, are proven to be significantly increased in schizophrenia [69, 70].

Homocysteine has also been suggested as a potentially useful biomarker of oxidative stress in schizophrenia. The harmful effect of homocysteine arises from generation of RS during its catabolism, which could oxidize membrane lipids and proteins, including enzymes. In fact, it has been suggested that homocysteine may have a significant influence on the development and clinical symptoms of schizophrenia [70-72].

Investigation of DNA damage can lead to additional information as a prospective indicator of carcinogenic and mutagenic potential. Elevated levels of 8-hydroxy-2-deoxyguanosine resulting from DNA damage were determined in hippocampus of post-mortem schizophrenia patients [38]. According to Halliwell *et al*., measurement of 8-hydroxy-2-deoxyguanosine is the most common method for evaluating DNA damage, however, artifactual results arising from isolation, preparation and analysis of DNA can easily occur [73]. One of the proposed approaches is to measure oxidative DNA damage in the intact cell. An assay using antibodies has been developed, but it is only semiquantitative. 8-hydroxy-2-deoxyguanosine can also be measured in urine, but it is not possible to establish its tissue origin [65, 73, 74].

Additionally, less specific biomarkers which may reflect total oxidant and antioxidant status of plasma (TOS and TAS, respectively) as well as oxidative stress index (OSI) calculated as ratio of TOS and TAS, have been used to evaluate oxidant-antioxidant balance in schizophrenia [51, 75, 76].

A summary of studies evaluating biochemical alterations related to oxidative stress in patients with schizophrenia is given in Table **1**.

Other potential biomarkers in schizophrenia, are less specific and include plasma arginase manganese blood platelets aggregability and apolipoprotein D level [22, 55, 77-79].

### Can Different Types of Schizophrenia be Distinguished by Measuring Peripheral Signs?

Since the distinction between the negative and positive symptoms is based mainly on behavioral criteria, and since most individuals with schizophrenia exhibit a mixed positive-negative symptomatology, additional physiological and biochemical criteria would be valuable. Available data show that potential biomarkers include RBC properties, antioxidant activity, lipid hydrolyzing enzymes such as phospholipase A2, and phospholipid and fatty acid composition. These properties not only differ between patients with predominantly negative and positive symptoms, but many times also correlate with the symptom severity [39]. Elevated levels of MDA and decreased PUFAs were found in patients with negative symptoms, indicating greater oxidative damage in this group of patients. Additionally, increased aggregation of RBC, O_2_^•− ^production and S100 B protein level, but decreased activity of GpX were associated with negative symptoms [12, 39, 44, 80-82]. On the other hand, positive symptoms are correlated with increased level of SOD [44].

Antioxidative status and lipid peroxidation have been studied in patients from different schizophrenia subgroups, such as disorganized, paranoid and residual schizophrenia. The total antioxidant response was found to be higher in the paranoid subtype than in disorganized, residual or undifferentiated subtypes [50]. SOD and GpX activities were significantly lower in paranoid and residual subtypes than in the disorganized subtype and the control group, while CAT activity was increased in all groups. The level of SOD in the residual group was higher than in the paranoid subtype [83]. Treatment refractory patients with schizophrenia suffered from greater lipid peroxidation and neuronal damage than non-refractory patients [84]. Gamma *et al*. studied the effects of clinical course of DSM-IV schizophrenia subtype on oxidative stress parameters. In comparison to deteoriated group, higher level of TBARS was associated with marked symptoms [85].

Although there is some empirical evidence that biomarkers of oxidative status correlate with symptoms in schizophrenia, there is not a clear correlation that could engage biomarkers in process of distinguishing different types of schizophrenia. Additional clinical studies are needed to further evaluate this theory.

## PATHOPHYSIOLOGY OF OXIDATIVE STRESS

### Dopamine Oxidation

The pathology of schizophrenia is suggested to be related to oxidative damage [7, 8, 51, 86, 87]. Oxidative stress plays a major role in the disease, even though it may not be the main cause.

Different mechanisms of oxidative stress in schizophrenia have been postulated. One of the most probable sources of RS is DA metabolism [7]. DA has a dihydroquinone structure and can be under physiological conditions non-enzymatically oxidized by molecular oxygen to form hydrogen peroxide (H_2_O_2_) and the corresponding *o*-quinone [6, 86, 88-90]. Non-enzymatic oxidation of catecholamines and PUFAs, combined with a deficient antioxidant system in the brain, may result in increased lipid peroxidation, which can influence the fluidity, integrity and permeability of membranes [87].

Oxidative deamination of DA by monoamine oxidase (MAO) can also lead to formation of H_2_O_2_ and 3,4-dihydroxyphenylacetaldehyde [91]. Consequently, both the auto-oxidation and the MAO-mediated metabolism of DA involve the formation of H_2_O_2_, a compound that can easily be reduced in the presence of ferrous iron (Fe^2+^) to form, through the Fenton reaction, hydroxyl radical (OH^•−^). Hydroxyl radical is considered to be a highly damaging free radical for living cells [6, 89-93]. Due to high iron content in the brain, DA auto-oxidation is probably the predominant source of elevated hydrogen peroxide concentration [89, 90].

Oxidative stress caused by elevated DA levels may enhance striatal glutamatergic neurotransmission leading to late-onset long-lasting permanent CNS damage [36].

There are also findings that NO may have a role in the pathophysiology of schizophrenia. NO is a gaseous neurotransmitter which is closely connected to dopaminergic and serotonergic neurotransmission. This provides a rationale for the involvement of NO and its pathway in schizophrenic disorders. Moreover, by interacting with O_2_^•−^, NO can produce peroxynitrite (ONOO^−^). This molecule can act as a neurotoxin by its interaction with the thiol groups on proteins as well as by decomposing into the highly reactive hydroxyl radical [21, 22, 77, 94-98].

### Mitochondrial Electron Transport Chain

Mitochondrial electron transport chain is an important source of RS. Mitochondria convert energy into adenosine triphosphate (ATP) by transport of protons across the inner mitochondrial membrane. The flow of electrons in the electron transport chain is a highly regulated process. The last step of this process is electron transport to oxygen molecule. Normally, oxygen is reduced to produce water. However, sometimes oxygen is prematurely and incompletely reduced to give the O_2_^•−^. This mechanism is well documented for Complex I and Complex III [6]. Inhibition of chain complexes I, III and IV induces a massive RS production. Complex I is the most sensitive and its inactivation by 16% results in a significant increase in RS [99]. There is evidence of abnormalities in the mitochondrial electron transport chain in an animal model and patients with schizophrenia [100-103].

### Genetic Factors

With the appearance of new technologies and exact phenotypic sub-classification, the recognition of genetic bases of psychiatric diseases and assessment of disease related alterations is bringing psychiatric research to a new level [104]. A genetic contribution to the pathogenesis of schizophrenia has been proposed [105-109]. There is evidence that an impaired capacity to synthesize GSH due to genetic polymorphism is a vulnerability factor for schizophrenia [23, 107]. It has been reported that GSH levels are decreased by 27% in the cerebrospinal fluid and 52% in prefrontal complex of drug-naive patients with schizophrenia [25]. Terpstra *et al*., and Matsuzawa *et al*., found reduction in GSH in brain, but the effect did not reach statistical significance [32, 34]. Additionally, Wood *et al*. conducted a study on first episode psychosis patients using magnetic resonance spectroscopy technique. Medial temporal lobe GSH concentrations were found to be 22% higher than those in control group. The authors concluded that the reason for the inconsistence with the results of the previous studies could be the difference in the stage of disease [33].

A positive association between schizophrenia and a functional polymorphism in the gene for manganese SOD has been shown in a sample of Japanese schizophrenia patients. A significant positive correlation between the total score on the Abnormal Involuntary Movements Scale (AIMS) and manganese SOD activity was found, which implies a general role of the oxidative stress-related genes in the pathogenesis of tardive dyskinesia (TD) [109]. Mukherjee *et al*. found that drug-naive first episode patients with schizophrenia have SOD activities significantly lower than those in healthy subjects, with no such differences in GpX and CAT activities [10]. In another study, in first-episode patients the level of SOD was significantly lower, while CAT was significantly higher and GpX was unchanged [82].

The molecular mechanisms of the effective antipsychotic drugs and recent advances in neural research suggest that several genes could be involved in the disorder. Examination of the polymorphisms of six genes, D2 and D3 DA receptors, serotonin A2 receptor, the brain-derived neurotrophic factor, ciliary neurotrophic factor and neurotrophin-3, showed that there are significant differences between patients with schizophrenia and a control group regarding the D2 receptor gene in all patients, and D2 and neurotrophin-3 in female patients. The authors conclude that the differences in clinical presentation of schizophrenia between genders could have a genetic background [105].

### Antipsychotic Treatment

It was suggested that the typical antipsychotic haloperidol, induces a six-fold increase in the levels of RS generated in mitochondria. The pyridinium ion metabolite derived from haloperidol might be cytotoxic, and is potentially related to drug induced extrapyramidal symptoms and cardiac functional disorder [45, 110-112]. Lipid peroxidation appears to be significantly higher after treatment with classical antipsychotics [45]. Moreover, it was found that atypical antipsychotics may improve oxidative status, increasing antioxidant levels and decreasing oxidative damage markers such as TBARS [82, 113]. Whether this is related directly with the effect of drugs on antioxidant enzymes and lipid peroxidation or whether the effect is indirect, through alteration in O_2_^•−^ and hydroxyl radical formation, remains to be determined [13, 55]. There appears to be a difference between typical antipsychotics, in terms of RS production after long-term treatment. Atypical antipsychotics show no change in lipid peroxidation product levels up to 90 days in treated rats. However, further treatment resulted in a significant increase in lipid peroxidation in the treatment with clozapine, ziprasidone and risperidone, but not with olanzapine [114]. Additionally, clozapine was found to induce oxidative stress and pro-apoptotic gene expression in neutrophils of patients with schizophrenia [79, 115]. Some authors indicate that the changes in the activity of antioxidant enzymes and levels of biomarkers of oxidative damage, studied in patients with schizophrenia, are independent of antipsychotic treatment and may reflect the pathophysiological process of the disease. No effects of duration of the disease, gender or dose of chlorpromazine equivalents were observed [21].

## THERAPEUTIC STRATEGIES FOR THE PREVENTION AND AMELIORATION OF OXIDATIVE DAMAGE

The endogenous antioxidant defense systems are not always entirely successful. The harmful effect in schizophrenia originating from RS could potentially be relieved by inactivation of RS by nutritional intake of antioxidants and essential fatty acids [11]. This is supported by an experiment on animal model. In rats fed with PUFAs brain SOD activity increased [37].

Vitamin E is a potent lipid soluble antioxidant that can stop the spread of the chain reaction in the lipid part of the cell membrane [18]. The concentration of vitamin E in peripheral nerves correlates with its concentration in plasma and depends on the dietary intake. Additionally, a deficit of vitamin E can attenuate the antioxidative defense [19]. Currently, there is not enough evidence that supplementation with vitamin E improves symptoms of neuroleptic-induced TD [116].

Vitamin C is a water soluble antioxidant that scavenges RS. It can inhibit peroxidation of the membrane phospholipids, and can also improve regeneration of vitamin E. There is a 10-times higher concentration of vitamin C in the brain than in serum. In its oxidized form, vitamin C can cross the brain blood barrier, presumably *via* glucose transporter GLUT1, and is retained in the brain tissue. It is hypothesized that increased dietary intake of vitamin C influences its concentration in the brain [19].

There are a few molecules with antioxidant activity, such as melatonin, α-lipoic acid and coenzyme Q10, that can pass the blood brain barrier and ameliorate oxidative status in rats. Their use in neurodegenerative diseases, evaluation of their antioxidant activity and possible effects on other systems need to be further characterized [19].

In studies on patients with schizophrenia, the most commonly used antioxidants were vitamins E and C. Vitamin E is a lipid soluble antioxidant able to prevent the oxidative damage. Nevertheless, it has a small potential in preventing oxidative damage to cytosolic proteins, mitochondria, and nucleus, where most of the RS are produced. Therefore, it is reasonable to add vitamin C, a water soluble antioxidant. The adjunctive use of vitamins C and E in schizophrenia requires caution since a high dietary intake will result in pro-oxidant rather than antioxidant actions [117]. In addition, supplementation with other antioxidants such as N-Acetyl Cysteine [118-121], rutin [122], Ginkgo biloba [54, 123-126], melatonin [127-129], hydroxytyrosol [130], caffeic acid phenethyl ester [131], resveratrol and quercetin [132] and lycopene [133, 134] has been suggested.

Among herbal supplemental antioxidative therapies used is schizophrenia, Ginkgo biloba was most widely studied. However, many studies were underpowered and poorly reported. Additionally, there is still some uncertainty which chemical compound was responsible for therapeutic effects. Two recently published meta-analyses [123, 126] demonstrated that treatment with Ginkgo biloba resulted in moderate improvement in total and negative symptoms of schizophrenia.

There is strong evidence that GSH concentration is decreased in cerebrospinal fluid, prefrontal cortex and postmortem caudate of patients with schizophrenia [23, 25, 33, 34, 107, 120]. Since cysteine is the rate-limiting precursor for GSH synthesis, it was proposed that treatment with N-acetyl cysteine could restore GSH levels [121]. A recent randomized multicenter study involving 84 subjects demonstrated a positive effect of N-acetyl cysteine on total, general and negative component of PANSS and revealed an improvement of CGI-severity and CGI-improvement scales. Additionally, treatment with N-acetyl cysteine was associated with an amelioration of akathisia. No significant effect on PANSS positive subscale was observed [118]. Bulut *et al*. reported a case of poorly responsive patient with schizophrenia, with significant improvement after supplementation with N-acetyl cysteine [119]. A growing body of evidence suggests potential effectiveness of N-acetyl cysteine in schizophrenia, which has to be further confirmed.

Since nutritional intake of essential PUFAs affects the fatty acid composition of the neuronal cell membrane phospholipids, the addition of PUFAs, particularly omega-3 and omega-6, could be a solution for the recovery of damaged membrane structures [37, 135-137].

The results of the use of vitamin E and omega-3 or 6- PUFAs are presented in Tables **2** and **3**. Only randomized, double-blind clinical trials were selected for evaluating the influence of supplemental treatment on clinical symptoms in schizophrenia. All accessible studies with vitamin C treatment and treatment with various combinations of antioxidants and PUFAs are presented.

To evaluate the effect of vitamin E treatment in patients with schizophrenia we performed a meta-analysis. Selection criteria for inclusion of the study were study duration (at least 4 weeks) and adequately reported treatment effect (difference in Abnormal Involuntary Movement Scale (AIMS) score compared to baseline value). Of the studies reported in Table **2** seven studies met the inclusion criteria [138-144]. From the extracted data weighted mean difference (WMD) with 95% confidence interval (95% CI) was calculated using Review Manager 4.2 (The Cochrane Collaboration, Oxford, UK). Random effect model was applied due to heterogeneity of the results of various studies. WMD of the improvement on AIMS was 1.92 (95% CI 0.60 to 3.24) suggesting significant treatment effect.

We find it interesting that two different studies of Adler *et al*., conducted with the same dose of vitamin E, had opposing results [138, 139]. The former study was conducted with a much smaller number of patients, 28 in comparison to 158 in the later study. Furthermore, the second study lasted much longer, 2 years in comparison to 12 weeks in the first study. In spite of this, the second study showed no significant influence of vitamin E on involuntary movement symptoms, as was observed in the first study. This suggests that there must be some difference in the chosen patient population. The authors themselves point out that when the first study was conducted, in 1993, therapy of schizophrenia was based mostly on the use of the classical antipsychotics while in 1999 atypical antipsychotics appeared in the prescribing patterns. Therefore, patients included in the later study were treated with classical or with atypical antipsychotics. Taking into account that there is considerable difference in the DA receptor blocking, the induction of RS production and in TD appearance between these two classes of antipsychotics, vitamin E would not be expected to have the same effect on symptoms improvement. The damaging potential and the symptoms observed differ considerably. Atypical antipsychotics induce much less TD in patients with schizophrenia. In spite of this, the Texas Medication Algorithm Project proposes that there is no reason to prefer atypical over classical antipsychotics in chronic schizophrenia, although opinion about this recommendation appears to be divided [145]. Considering the fact that classical antipsychotics are still used in therapy of schizophrenia, further studies evaluating supplementation with vitamin E are recommended. Both studies by Adler *et al*. included very few female patients, making it a highly unrepresentative patient sample.

As far as vitamin C is concerned, there is very little data. Available studies with vitamin C treatment and treatment with combinations of vitamin C and vitamin E show a significant improvement in BPRS score and reduction in dyskinetic movement total score [117, 146].

From the review of the effects of supplementation with PUFAs in schizophrenia, it can be concluded that they might improve mental state and movement disorder symptoms. This is mostly associated with omega-3 PUFAs, since there is only limited information on supplementation with omega-6 PUFAs [147]. Meta-analysis of 5 eligible studies reported in Table **3** [148-152] revealed insignificant improvement on PANSS score (WMD 2.85 95% CI -1.20 to 6.89). There are still too few data on the role of essential fatty acid supplementation in the treatment of patients with schizophrenia, and larger, well designed clinical studies need to be conducted.

Despite the suggestion that combined therapy with antioxidants and PUFAs is potentially more effective than monotherapy in patients with schizophrenia [3], only two studies with combined treatment were found [49, 153]. Positive results were observed in both of the studies which, however, were not controlled.

## CONCLUSIONS

There is a growing body of evidence that oxidative stress is involved in the pathology of schizophrenia. Mechanisms involved include genetic susceptibility, catecholamine metabolism, and critical surroundings in the CNS (glutamate, iron, highly oxidizable fatty acids). Antipsychotic treatment, especially treatment with classical antipsychotics, also contributes to oxidative stress promotion.

Oxidative stress in schizophrenia can be evaluated with a wide spectrum of biomarkers. However, specific biomarkers based on a non-invasive sampling method, stable in biological materials, and easy to measure, has still to be discovered. It is suggested that measurements in blood can reflect oxidative status in CNS and can be clinically useful in identifying patients at high risk of developing adverse effects. However, this needs further confirmation. There are indications that different types of schizophrenia, and positive and negative symptoms, can be distinguished by the levels of biomarkers. This would be of great help in the diagnosis of schizophrenia and in predicting the course of disease. Attempts to ameliorate oxidative damage and therapeutically induced extrapyramidal syndrome are of considerable importance. The use of antioxidants and PUFAs in the treatment of schizophrenia has yielded some positive results, but still remains experimental. There is a need for larger, well designed clinical studies. Further studies need to be placebo-controlled with larger numbers of patients on standardized antipsychotic therapy and duration of disease. Since PUFAs administered together with antioxidant vitamins are potentially effective low cost supplemental treatment with few side effects, such therapy may become a significant advance in the treatment of schizophrenia.

## Figures and Tables

**Table 1.** A Summary of Studies Evaluating Biochemical Alterations Related to Oxidative Stress in Patients with Schizophrenia

Biomarker	Decreased Level	Increased Level
**Antioxidants**		
**SOD**	Akyol *et al*. 2002, Ben Othmen *et al*. 2008, Dietrich-Muszalska *et al*. 2005, Li *et al*. 2006, Mukerjee *et al*. 1996, Pavlovic *et al*. 2002, Ranjekar *et al*. 2003, Zhang *et al*. 2006, Zhang *et al*. 2007	Abdala *et al*. 1986, Aluntas *et al*. 2000, Dakhale *et al*. 2004, Kuloglu *et al*. 2002, Michel *et al*. 2004, Rukmini *et al*. 2004, Surapaneni 2007
**GpX**	Abdala *et al*. 1986, * Akyol *et al*. 2002, Aluntas *et al*. 2000, Ben Othmen *et al*. 2008, Li *et al*. 2006, * Mukerjee *et al*. 1996, Zhang *et al*. 2006, Zhang *et al*. 2007.	Kuloglu *et al*. 2002, Surapaneni 2007
**CAT**	Ben Othmen *et al*. 2008, Li *et al*. 2006, * Mukerjee *et al*. 1996, * Pavlovic *et al*. 2002, Ranjekar *et al*. 2003, Surapaneni 2007, Zhang *et al*. 2006, Zhang *etal*	Rukmini *et al*. 2004
**GSH**	Aluntas *et al*. 2000, Dadheech *et al*. 2006, Pavlovic *et al*. 2002, Surapaneni 2007, Yao *et al*. 2006, Dietrich-Muszalska *et al*. 2009	
**GR**	Yao *et al*.2006	
**Uric acid**	Yao *et al*.1998	
**Vitamin E**	Dadheech *et al*. 2006, Surapaneni 2007	
**Vitamin C**	Dadheech *et al*. 2006, Surapaneni 2007	
**Total antioxidant status (TAS)**	*Pazvantoglu *et al*. 2009, Ustundag *et al*. 2006, Virit *et al*. 2009	
**Total oxidant status (TOS)**	* Pazvantoglu *et al*.2009, *Virit *et al*. 2009	
**TBARS (MDA)**		Akyol *etal*. 2002, Aluntas *et al*. 2000, Ben Othmen *et al*. 2008, Dadheech *et al*. 2006, Dakhale *et al*. 2004, Dietrich-Muszalska *et al*. 2005, Khan *et al*. 2002, Kropp *et al*. 2008, Kuloglu *et al*.2002, Pavlovic *et al*. 2002, Ranjekar *et al*. 2003, Rukmini *et al*. 2004, Zhang *et al*. 2006, Zhang *et al*. 2007
**PUFAs**	Ranjekar *et al*. 2003, Khan *et al*. 2002	
**Lipide peroxide**		Li *et al*. 2006
**3-Nitrotyrosine**		Dietrich-Muszalska *et al*. 2009, Dietrich-Muszalska and Olas 2009c.
**Carbonyl groups**		Dietrich-Muszalska*et al*. 2009
**8-hydroxy-2-deoxyguanosine**		Nishioka and Arnold 2004
**Reactive species production**		
**Xantine oxidase**		Akyol *et al*. 2002
**Homocysteine**		Akanji *et al*. 2007, Dietrich-Muszalska *et al*. 2009
**Superoxide radical release**		Sirota *et al*. 2003
**NO**		Akyol *et al*. 2002, Li *et al*. 2006, Yilmaz *et al*. 2007, Yanik *et al*. 2003

(CAT – catalase, GpX - Glutathione peroxidase, GR - glutathione reductase, GSH – glutathione, MDA – malondialdehyde, NO – nitric oxide, PUFAs - polyunsaturated fatty acids, SOD - Superoxide dismutase, TBARS - thiobarbituric acid related substances)

* No change in biomarker level

**Table 2.** Effect of Supplemental Treatment (Vitamin E and C) in Patients with Schizophrenia, Observed in Double-Blind Randomized Studies. Reports for Vitamin E were Included According to Data from Cochrane Library Data Base (Soares and McGrath, 2001)

Vitamin E Studies	Study Duration, Number of Patients	Intervention	Outcome
**Adler 1993 **	12 weeks, N=28	Vitamin E 1600 IU/day	Significant reduction of AIMS score in favor of vitamin E
**Adler 1999 **	2 years, N=158	Vitamin E 1600 IU/day	No significant reduction of AIMS and BPRS
**Dabiri 1994 **	12 weeks, N=12	Vitamin E 1200 IU/day	Significant reduction of AIMS
**Elkashef 1990 **	4 weeks, N=10	Vitamin E 1200 IU/day	Significant reduction of AIMS
**Lam 1994 **	6 weeks, N=16	Vitamin E 1200 IU/day	No significant reduction of AIMS
**Lohr 1996 **	8 weeks, N=55	Vitamin E 1600 IU/day	Significant reduction of AIMS and not of BPRS
**Sajjad 1998**	7 months, N=20	Vitamin E 600 IU/day	Significant reduction of AIMS score
**Vitamin C studies**
Nikolaus 2002*(Nicolaus *et al*., 2002)	2 years , N=6	Vitamin C 200 mg/day and vitamin E 1.8 mg/day	Significant reduction in dyskinetic movements total score
**Dakhale 2005** (Dakhale *et al*. 2005)	8 weeks , N=40	Vitamin C 500 mg/day	Significant reduction in MDA and BPRS

* Prospective open study.

(N- number of subjects, AIMS- Abnormal Involuntary Movement Syndrome Scale, BPRS - Brief Psychiatric Rating Scale, TDRS - Tardive Dyskinesia Rating Scale, MDA - Malondialdehyde).

**Table 3.** Effect of Supplemental Treatment with Polyunsaturated Fatty Acids or Combination of Antioxidants and Polyunsaturated Fatty in Patients with Schizophrenia, Observed in Double Blind Randomized Studies. Reports for Fatty Acid Supplemental Therapy were Included According to Data from Cochrane Library Data Base (Joy *et al*., 2007)

Fatty Acids Studies	Method	Intervention	Outcome
Emsley 2006	12 weeks, N=77	E-EPA 2g/day	No significant difference in ESRS
Emsley 2002	12 weeks, N=40	E-EPA 3g/day	Significant reduction in PANSS scores
Fenton 2001	16 weeks, N=90	E-EPA 500 mg/day and vitamin E	No significant change in PANSS, M-ADRS, AIMS, S-ARS, CGI
Peet 2001	12 weeks, N=55	EPA 2g, DHA 2g Comparative study	Significant reduction in PANSS scores. EPA is superior to DHA
Peet 2002	12 weeks, N=55	EPA 1g/day, EPA 2g/day, EPA 3g/day, EPA 4g/day. Comparative study	Significant reduction in PANSS scores, the biggest for those who had EPA 2g/day
Vitamins E and C and fatty acids			
Arvindakshan 2003 *	4 months, N=33	EPA/DHA 180:120 mg Vitamin E:C 400 IU/bid: 500mg/bid	Significant reduction of PANSS and BPRS and increase of QOL
Sivrioglu 2007 *	4 months, N=17	EPA/DHA 180:120 mg Vitamin E:C 400 IU/bid: 1000mg/day	Significant reduction of BPRS, SANS, S-ARS and BARS.

* Prospective open study.

(N- number of subjects, ESRS -Extrapyramidal Symptom Rating Scale, AIMS - Abnormal Involuntary Movement Syndrome scale, BPRS - Brief Psychiatric Rating Scale, PANSS - Positive and Negative Syndrome Scale, SANS - Scale for Assessment of Negative Symptoms, BARS - Barnes Akathisia Rating Scale, M-ADRS - Montgomery-Asberg Depression Rating Scale, S-ARS - Simpson-Angus Rating Scale, CGI - Clinical Global Impression scale, QOL - Henrich’s Quality of Life scale, EPA - Eicosapentaenoic Acid, E-EPA -Ethyl eicosapentaenoic acid, DHA - Docosahexaenoic acid).
